# DEVO: an ontology to assist with dermoscopic feature standardization

**DOI:** 10.1186/s12911-023-02251-y

**Published:** 2023-08-18

**Authors:** Xinyuan Zhang, Rebecca Z. Lin, Muhammad “Tuan” Amith, Cynthia Wang, Jeremy Light, John Strickley, Cui Tao

**Affiliations:** 1 School of Biomedical Informatics, The University of Texas Health Science Center at Houston, Houston, TX USA; 2 Division of Dermatology, Washington University School of Medicine, St. Louis, MO USA; 3 Department of Information Science, University of North Texas, Denton, TX USA; 4 Department of Biostatistics and Data Science, University of Texas Medical Branch, Galveston, TX USA; 5 Department of Internal Medicine, University of Texas Medical Branch, Galveston, TX United States

**Keywords:** Ontology, Dermatology, Dermoscopic features, Metaphoric terminology

## Abstract

**Background:**

The utilization of dermoscopic analysis is becoming increasingly critical for diagnosing skin diseases by physicians and even artificial intelligence. With the expansion of dermoscopy, its vocabulary has proliferated, but the rapid evolution of the vocabulary of dermoscopy without standardized control is counterproductive. We aimed to develop a domain-specific ontology to formally represent knowledge for certain dermoscopic features.

**Methods:**

The first phase involved creating a fundamental-level ontology that covers the fundamental aspects and elements in describing visualizations, such as shapes and colors. The second phase involved creating a domain ontology that harnesses the fundamental-level ontology to formalize the definitions of dermoscopic metaphorical terms.

**Results:**

The Dermoscopy Elements of Visuals Ontology (DEVO) contains 1047 classes, 47 object properties, and 16 data properties. It has a better semiotic score compared to similar ontologies of the same domain. Three human annotators also examined the consistency, complexity, and future application of the ontology.

**Conclusions:**

The proposed ontology was able to harness the definitions of metaphoric terms by decomposing them into their visual elements. Future applications include providing education for trainees and diagnostic support for dermatologists, with the goal of generating responses to queries about dermoscopic features and integrating these features to diagnose skin diseases.

**Supplementary Information:**

The online version contains supplementary material available at 10.1186/s12911-023-02251-y.

## Background

The utilization of dermoscopic analysis is becoming more and more important for the diagnosis of skin diseases by physicians and possibly even artificial intelligence. Dermoscopy is a non-invasive, in vivo technique primarily used to examine pigmented anatomic skin lesions [1–4] and identify features that may not be visible to the naked eye. It has been shown to facilitate the clinical recognition of several inflammatory and infectious diseases [5] and significantly improve the clinician's diagnosis of pigmented and non-pigmented skin lesions, including skin cancers and inflammatory and infectious diseases [3, 6–8]. However, the utilization of dermoscopy requires additional training for dermatologists, including the ability to recognize specific dermoscopic features. To master the skill of dermoscopic analysis, dermatologists need to learn designated terminology and diagnostic rules developed based on content experts.

With the expansion of dermoscopy in recent years, its vocabulary has proliferated, but the rapid evolution of the vocabulary of dermoscopy without standardization leads to confusion and redundancy. Two competitive terminologies exist: one is a metaphoric terminology that includes numerous metaphors, such as ‘‘leaf-like’’ areas; the other is a descriptive terminology based on five basic elements [9], such as lines (more specifically, “thick or reticular lines that vary in color”). Even though the metaphoric terminology is easier to understand, the analogies provided may be ambiguous, redundant, or even harmful, as poorly defined metaphors lend themselves to misinterpretation. This creates barriers for research, education, and clinical care. Hence, this is the motivation for creating a descriptive language with a simple and logical structure. However, when dealing with complicated structures, descriptive terminology could result in a long and cumbersome expression, especially in comparison with the corresponding metaphoric expression. Since both terminologies have their advantages and disadvantages, this reveals the need to establish a dictionary that harmonizes and standardizes the existing terms. The third consensus conference conducted by the International Society of Dermoscopy tried to provide a dictionary that maps between both terminologies [10], but problems still exist. We found some characteristics defined by metaphoric terms not covered by the descriptive terminology. Furthermore, some basic terms defined by the descriptive terminology are not straightforward, creating obstacles for the trainee or computer to learn. These issues encourage the development of an ontology that defines consistent terms to benefit knowledge exchange in this domain.

Ontologies are software artifacts that represent domain knowledge using terminologies and taxonomic relations to express domain meaning. By describing and linking concepts together using taxonomic relations, they can explicitly describe grounded knowledge that can be shared with agents to facilitate common understanding. Knowledge representation is one of the essential components of artificial intelligence, alongside vision and inferencing [11], which then elicits opportunities to analyze domain knowledge. In addition, ontologies are encoded with semantic machine-level syntax (e.g., OWL [12] and RDF [13, 14]) that can mimic first order logic, thereby producing machine-based inferences from the ontology with reasoner software, like HermiT [15] or Pellet [16].

In the dermoscopy domain, the language of dermoscopy is technical because of its specific vocabulary that is incomprehensible outside its context. Hence, this is the motivation for creating a definitive language with a simple and logical structure. This proposed language could break down the characteristics of a “leaf-like area” into individual categories, such as color, pattern, and texture, so that any user of the dermoscopy terminology can have a clearer and more standardized understanding for the same terms. Using an ontology to describe domain knowledge can not only help to provide a way to reuse domain knowledge, but also provide an encoding of knowledge that machines can understand and automate large-scale machine processing.

Before designing the ontology, we searched for the related skin disease ontologies in distributed data sources, especially disease ontologies in BioPortal [17]. There have been repeated initiatives trying to develop ontologies for skin disease, some dedicated to skin disease categories and some diving further into phenotypes [18]. Some ontologies were also trying to take into consideration expert knowledge such as the ABCD rule, which involves four characteristics of a lesion to help with computer-aided diagnosis systems [19–21]. However, none of these ontologies could be utilized to help both humans and computers better understand dermoscopic features, as they did not deconstruct these features into basic elements that can be visualized by computers. Dermoscopic features are the foundation for all diagnostic rules, including the ABCD rule. If a computer is able to detect and understand skin images under a dermoscopic-feature level, then it can help classify not just the benign. Vs melanoma skin disease category, but all the skin categories under diagnostic rules. Hence, in order to fill this gap, we planned to build an object-oriented, top-down developed skin disease feature ontology based on the results of the consensus from the International Society of Dermoscopy using Protégé [22].

Our approach to develop an ontology for dermoscopy involved two phases. The first phase was to create an lower-level ontology that covers the basic aspects and elements in describing visualizations, such as shapes and colors. The second phase was to create a domain ontology that harnesses the lower-level ontology to formalize the definitions of dermoscopic metaphorical terms. The aforementioned lower-level ontology is called the Elements of Visuals Ontology (EVO), and the domain level ontology is called the Dermoscopy Elements of Visuals Ontology (DEVO). By formally representing information about dermoscopic features for skin lesions, DEVO can not only help with better understanding and interpretation of domain knowledge, but also provide a way to reuse domain knowledge.

## Methods

### Development of EVO

**Fig. 1:**
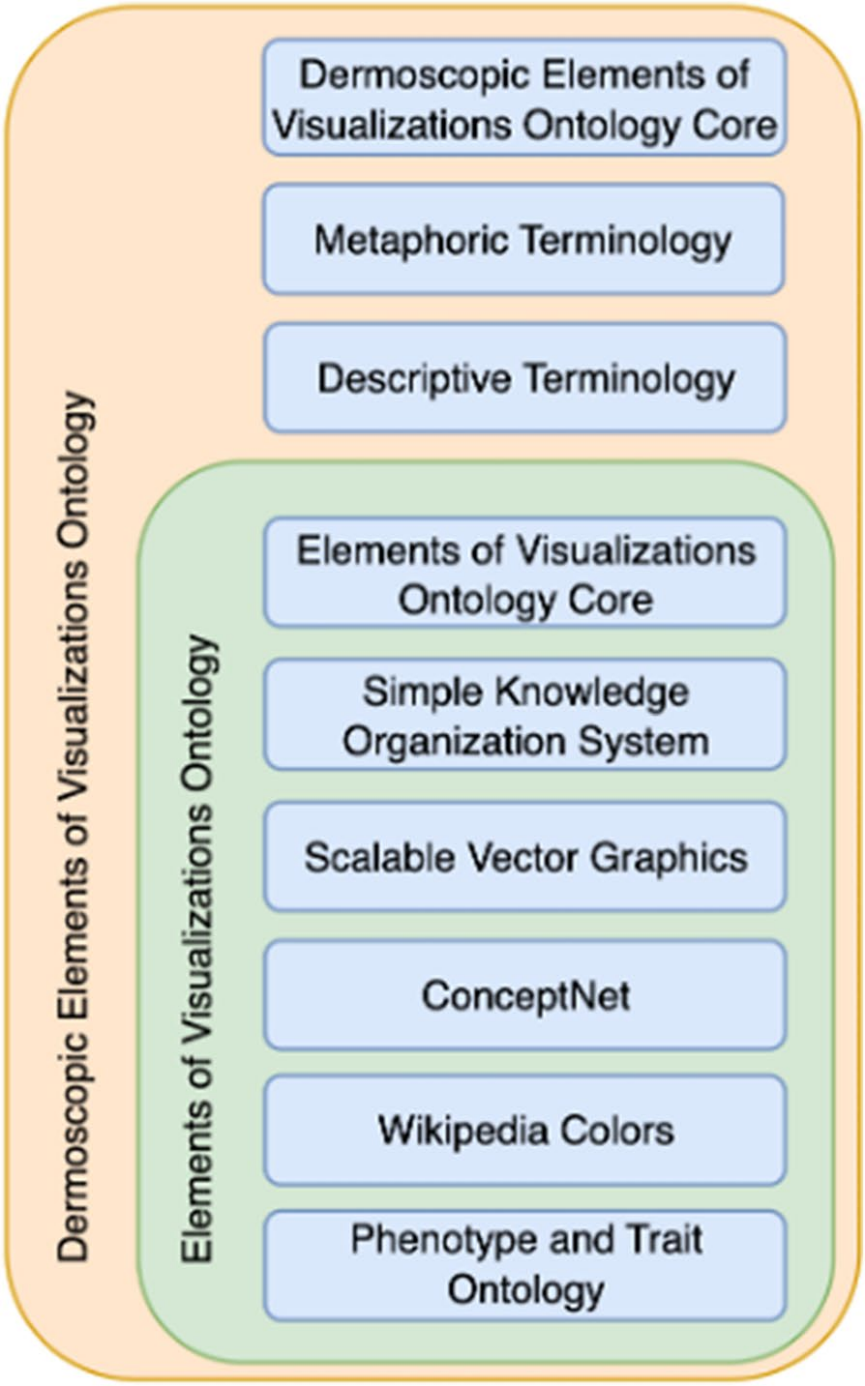
Conceptual stack of the Dermoscopy Elements of Visuals Ontology (DEVO) and Elements of Visuals Ontology (EVO) describing the composition of their main concepts and their relationships

We first created a foundational ontology called the Elements of Visuals Ontology (EVO). It provides a set of concepts and taxonomic structures that aims to decompose visuals to basic elements and conceptualize these elements to provide meaning. Based on EVO, we developed the Dermoscopy Elements of Visuals Ontology (DEVO) to further decompose technical dermoscopic terminologies. The relationship of EVO and DEVO is shown in Fig. 1.

The basic premise of this work is predicated on the notion that every visualization is a composite of visualization elements. These elements, based on some of the SVG vocabulary, include Shape, Color, Pattern, Stroke, etc. The total expression of the visualization is built on these elements.

**Definition 1** Given the gamut of visualization elements, we define these elements VE as a set of any number of shapes S, textures T, colors C, patterns P, spatial patterns SP, strokes SK, points PT, spatial relationships SR, sizes SZ, and Paths PH.

1 $$VE \ni \{{\varvec{S}}, {\varvec{T}},{\varvec{C}},{\varvec{P}},{\varvec{S}}{\varvec{P}},{\varvec{S}}{\varvec{K}},{\varvec{P}}{\varvec{T}},{\varvec{S}}{\varvec{R}},{\varvec{S}}{\varvec{Z}},\boldsymbol{ }{\varvec{P}}{\varvec{H}}\}$$

where ${\varvec{S}}, {\varvec{T}},{\varvec{C}},{\varvec{P}},{\varvec{S}}{\varvec{P}},{\varvec{S}}{\varvec{K}},{\varvec{P}}{\varvec{T}},{\varvec{S}}{\varvec{R}},{\varvec{S}}{\varvec{Z}},\boldsymbol{ }{\varvec{P}}{\varvec{H}}$ are sets of finite numbers of shapes S, textures T, colors C, patterns P, spatial patterns SP, strokes SK, points PT, spatial relationships SR, sizes SZ, and Paths PH respectively.

**Definition 2** Furthermore, with the set of visualization elements, we define our description of visualization VZ as a composite of visualization elements VE. For example, a visualization can be just one shape, or a pattern, an individual color, or an aggregate of a shape with a color, or multiple shapes comprising a pattern, etc.

2 $$\forall VZ \models {VE}_{1}\curlywedge {VE}_{2}\curlywedge \cdots \curlywedge {VE}_{n}$$

where n denotes the maximum number of VE.

**Fig. 2:**
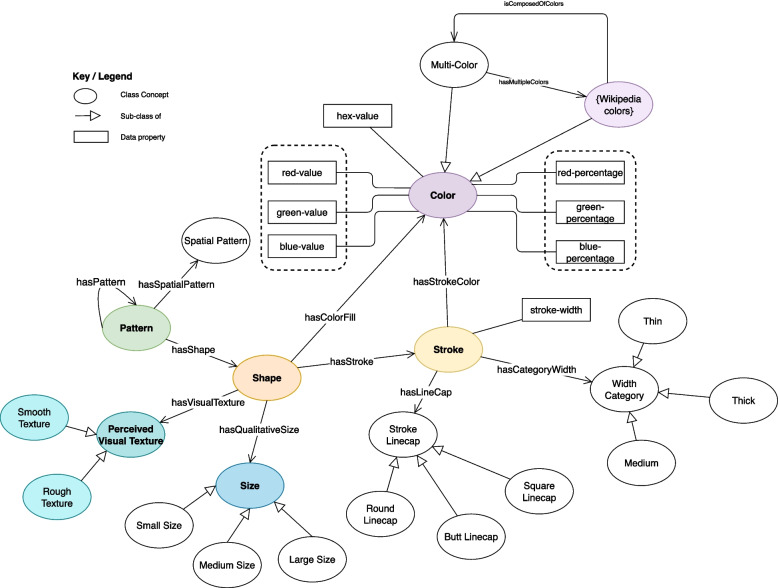
General concepts of visualizations for EVO

Figure 2 has shown the essential concepts of visualization elements. The construction of this model is largely informed by the SVG standards [23]. In essence, the Pattern class serves as a container to Shape, along with the usage of Stroke and Color. We provided a detailed description for the Pattern class in definition 3.

**Definition 3** A pattern is a composite of shapes (Pattern > hasShape > Shape), and can also be comprised of one or more patterns (Pattern > hasPattern > Pattern). We define a pattern P that is a set of shapes S or equally a set of patterns P with an associated spatial pattern SP.

3 $$\forall P =\left\{{S}_{1}\curlywedge {S}_{2}\curlywedge \cdots \curlywedge {S}_{n}\right\}=\{{P}_{m}\curlywedge SP\}$$

where n denotes maximum number of shapes S, ${P}_{m}$ is a set of patterns P.

For certain circumstances, the model utilizes the Spatial Pattern concept, influenced by Phenotype And Trait Ontology (PATO)’s [24] own spatial pattern. The Spatial Pattern describes the overall arrangement of the components within the Pattern using the hasSpatialPattern object property.

Also, this model elaborates on the Shape concept. Each Shape has a color fill (Shape > hasColorFill > Color), and Stroke (Shape > hasStroke > Stroke) which itself has its own color description (Stroke > hasStrokeColor > Color). Like most of the concepts from EVO, the Color concept inherits some of the features present in the SVG model. EVO’s Color concept contains a variety of color subclasses with RGB and hex-value data properties, derived from a list sourced from Wikipedia [25].

Aside from the color data, we also created a concept called Multi-Color to accommodate a mix of several colors. This concept relates to the subclassed Colors through the hasMultipleColor object property (Multi-Color > hasMultipleColors > Color.[Color]). An inverse of hasMultipleColor is also provided with isComposedOfColors (Color.[Color] > isComposedOfColors > MultiColor).

For each Shape, we defined Stroke according to SVG, which is “the operation of painting the outline of a shape or the outline of character glyphs in a text string” [23]. Essentially, Stroke serves as an outline of Shape, like a border with property options. EVO provides an integer data property called stroke-width to indicate the relative thickness of a border. Similarly, we included a qualitative width with three categories of Thin, Thick, and Medium.

We also utilized the Size concept to describe the relative size of visual elements based on the terminologies from ConceptNet and PATO (2-D extent from size). Similarly, Perceived Visual Texture, used to describe texture base on sight, leverages concepts from PATO and ConceptNet with *Smooth Texture* and *Rough Texture* types.

4 $$\forall S \models hasColorFill(S, C)\curlywedge hasStroke(S, SK)$$

$$\curlywedge hasVisualTexture(S, T) \curlywedge hasSize(S, SZ)$$

**Definition 4** Provides a general model of the Shape concept, where we define shape S having a color fill hasColorFill for color C, having a stroke element hasStroke for stroke SK, having a texture hasVisualTexture for perceived visual texture T, and having a size description hasQualitativeSize for size SZ.

Our model also addresses the expression of adjacency between multiple *Shape* concepts. We defined the category “Spatial relationships” to explore how shapes are spatially distributed, utilizing the Dimensionally Extended 9-Intersection Model (DE-9IM), a mathematical standard, to denote spatial placement of objects [26]. According to the DE-9IM standard, the spatial relationships are defined by the following properties:

- intersection of the boundary where the boundaries of two objects are shared.
- common interior parts where the interior regions of objects are shared.
- boundary as part of the interior, where the boundary of one object subsumes the interior of another object.
- and the interior as part of the boundary, where the interior of an object subsumes the boundary of another object.

**Fig. 3:**
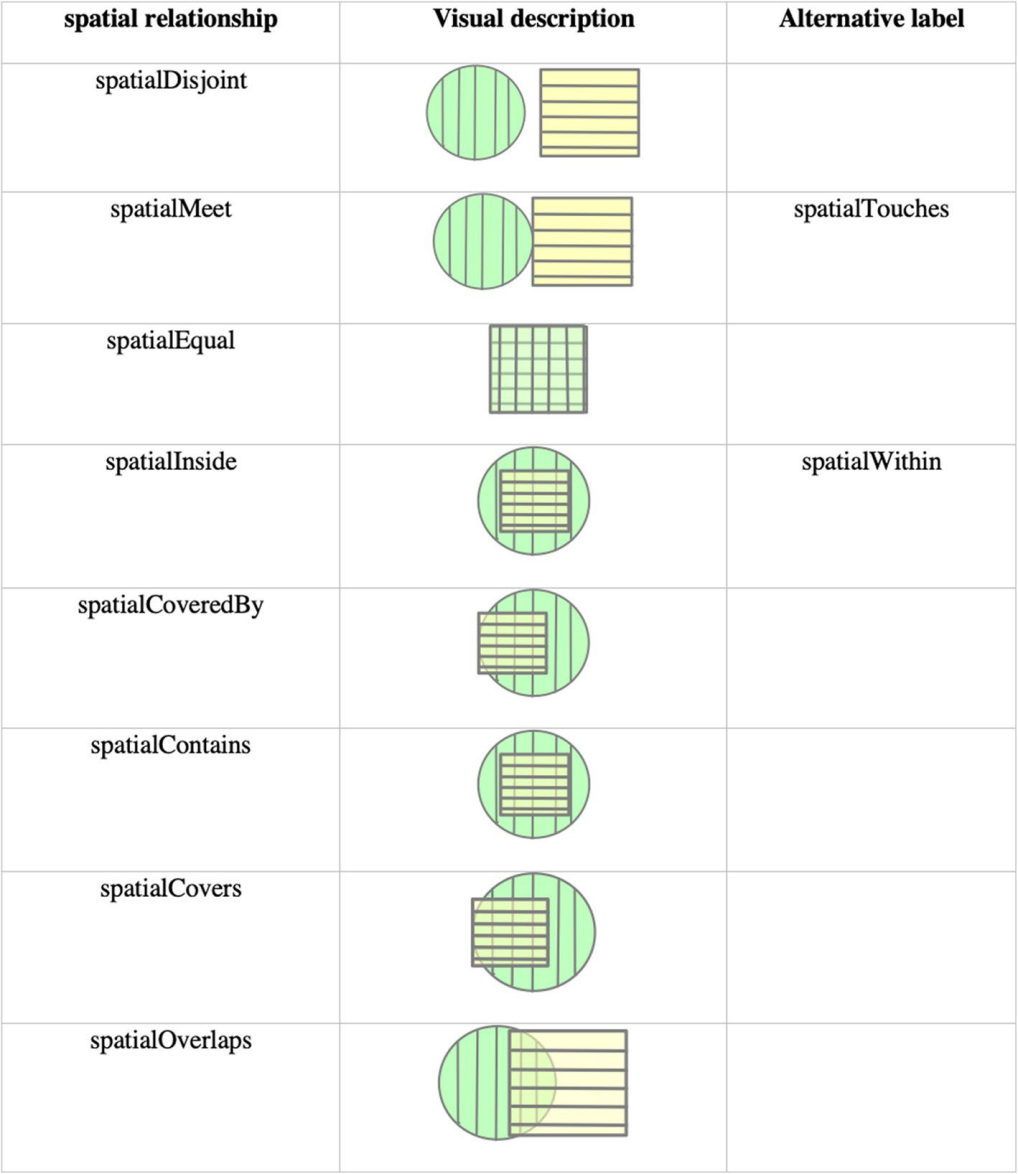
Spatial relationship description for EVO

For two objects, the object property *spatialDisjoint* is defined as no intersection of the boundary, no common interior parts, no boundary as part of the interior, and no interior as part of the boundary. In contrast, *spatialOverlap* is the opposite where there is intersection of the boundary, common interior parts, the boundaries as part of the interior, and the interior is part of the boundary. *spatialMeet* is defined as only having an intersection of the boundary. spatialEqual is described as having an intersection of the boundary and common interior parts. *spatialInside* is based on common interior parts and boundary as part of the interior. *spatialCoveredBy* and *spatialCovers* share the property of having an intersection at the boundary and common interior parts, but where they differ is the boundary's relationship with the interior. For *spatialCovers*, object A is on top of object B, so the interior of A is part of the boundary. For *spatialCoveredBy*, where object A is below object B, the boundary of B is part of the interior. Lastly *spatialContains* is when there is intersection of the boundary and common interior parts, where the interior is part of the boundary. Fig. 3 outlines each spatial relationship with a visual description.

**Fig. 4:**
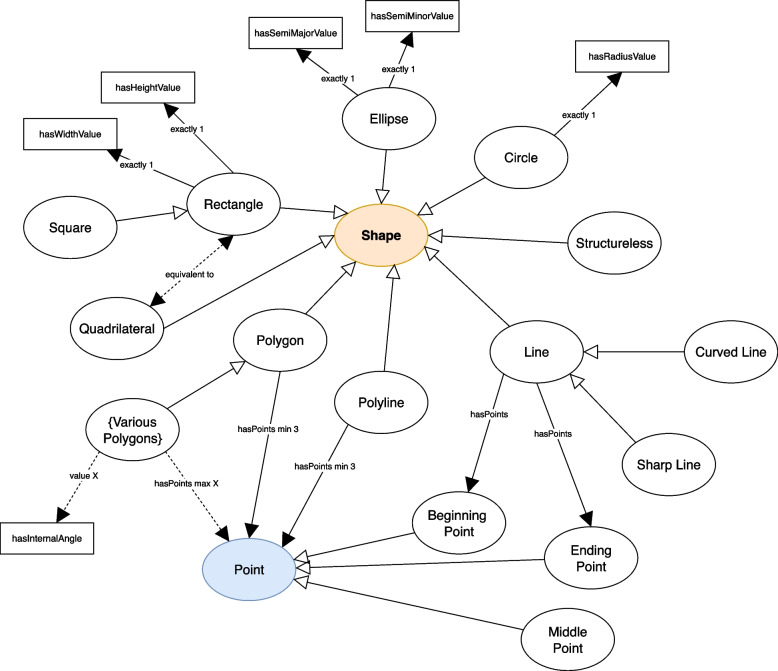
Shapes concept from EVO

The *Shape* concept is further elaborated with subclasses of *Shape* (shown in Fig. 4). *Circle*, *Ellipse, Polygon, Polyline, Line,* and *Rectangle* are all based on the SVG standard [23], “A graphics element that is defined by some combination of straight lines and curves. Specifically: ‘circle’, ‘ellipse’, ‘line’, ‘path’, ‘polygon’, ‘polyline’ and ‘rect’ (Rectangle).” We further extended the subclasses with additional shapes, including adding a *Structureless* concept for undefined shapes. For most of the *Shape* concepts, their basic definition includes *Points*. EVO includes a *Point* concept that is used to define some basic structures of shapes. For example, a triangle has exactly 3 points (e.g. vertices) as its structural definition. The *Point* concept is further subclassed as *Beginning Point* and *Ending Point* to define a point at a starting section of a visualization and at a closing section. There is also the subclass of *Middle Point* that covers points that may exist between *Beginning Point* and *Ending Point*.

### Evaluation of EVO

We used Hootation [27], a natural language generation tool that can produce equivalent natural language statements for ontologies, to evaluate the logical axioms of EVO. For example, the axiom, Ellipse ⊑ Shape from EVO would produce the sentence “every ellipse is a shape.” Three reviewers: RL, XZ, and TA independently reviewed the sentences, and we calculated pairwise percentage agreement to assess agreement of the independent evaluations. In addition, we utilized the evaluations to revise the ontology based on the independent review.

Each of our evaluators were given 182 natural language statements extracted from the ontology through Hootation. All three evaluators had previous research experience in ontology development and were thus suited to evaluate the translated axioms. The evaluators were instructed to denote "Y" if they agreed with the logical axiom's expression in natural language, or "N" or "X" if they did not agree or were not sure of the statement's veracity.

### Development of DEVO

We designed EVO to serve as a foundation ontology to describe the basic elements of visualizations. In the next phase of our approach, we used the concepts and structure of to develop the domain ontology DEVO (Dermoscopy Elements of Visuals Ontology), which further enriches the EVO model with concepts pertaining to the field of dermoscopy.

A comprehensive list of metaphoric definitions was generated during the third consensus conference of the International Society of Dermoscopy[10], so we aimed to translate these terms into machine-intelligible descriptive language for DEVO. For each metaphoric term in the list, we discerned the visual elements present in the definition, enabling us to create comprehensive diagrams to aid in the development of our ontology.

**Definition 5** Using EVO as a foundation, we defined visual elements VE as a set of any number of shapes S, textures T, colors C, patterns P, spatial patterns SP, strokes SK, points PT, spatial relationships SR, size SZ, relative position RP*, and body position BP*.

*denotes any new visual element that was not present in EVO

5 $$VE=\left\{{S}_{n}, {T}_{n},{C}_{n},{P}_{n},{SP}_{n},{SK}_{n},{PT}_{n},{SR}_{n},{SZ}_{n}{,PH}_{n},R{P}_{n}^{*},B{P}_{n}^{*}\right\}$$

where ${S}_{n},{T}_{n},{C}_{n},{P}_{n},{SP}_{n},{SK}_{n},{PT}_{n},{SR}_{n},{SZ}_{n}{,PH}_{n},R{P}_{n}^{*}, B{P}_{n}^{*}$ represent the corresponding elements sets.

Shape (S)

The five basic elements that comprise the descriptive terminology are: circles, lines, dots, clods, and pseudopods [9]. An area lacking basic elements is classified as structureless. In light of these basic elements, we chose to integrate the following *Shape* subclasses from EVO into DEVO: *Circle*, *Line, Structureless,* and *Polygon*. Of these, *Circle*, *Line,* and *Structureless* are directly analogous to the basic elements in the descriptive terminology. Within the *Line* subclass, we also differentiated between *Sharp, Curved, Serpiginous, Wavy, Dotted, and Double Lines* (if unspecified, a line was assumed to be straight)*.* Although not one of the basic elements described by Kittler, we also included the shape *Polygon* from EVO since it was mentioned in the *Cobblestone* pattern definition [10].

While we did not add specific shapes termed “dots,” “clods,” or “pseudopods,” we still thought it important to incorporate all of Kittler’s basic elements in DEVO. For instance, we chose to define a “dot” as simply a circle of a small size. Additionally, as it is unlikely that any skin lesion would be a perfect circle, we decided to loosely define *Circle* as a rounded shape that may not have uniform radius for maximal clinical utility. Thus, a circle in DEVO would also encompass the terms “clod,” “globule,” “ellipse,” etc. Since we are trying to create machine-intelligible definitions, another reason why we did not add the “clod” basic element is because the word “clod” itself is a metaphor that refers to a clod of earth [28], which is less straightforward to understand than the word “circle.” Holes, follicle openings, and adnexal openings were all classified as types of circles in DEVO. Finally, since we defined a pattern as a composite of shapes (Definition 3), “pseudopod” is actually a pattern, not a shape, since it contains peripherally-located circles (*Circle* > hasRelativePosition > *Periphery*) meeting radial lines (*Line* > hasSpatialRelationship > *Radial Spatial Relationship*; *Circle* > spatialMeets > *Line*). Another pattern would then use the object property hasPattern instead of hasShape to note that it contains pseudopods (e.g., *Starburst Pattern* > hasPattern > *Pseudopods*).

Texture (T)

We extended the Perceived Visual Texture concept from EVO to include Shiny Texture for the Milia-Like Cyst (Cloudy/Starry) patterns in DEVO. No other textures were mentioned in the pattern definitions.

Color (C)

Colors integrated from EVO into DEVO include White, Pink, Red, Orange, Yellow, Bright Yellow, Green, Blue, Blue White, Blue Gray, Indigo, Violet, Brown, Light Brown, Gray Brown, and Black. Additionally, we extended the Color concept from EVO to include Non-pigmented Color, Hypopigmented Color, Pigmented Color, Dark Color, Uniform Color, and Variable Color in DEVO. However, it is important to keep in mind that these color subclasses depend on the individual’s skin color (Hypopigmented is lighter than their skin color, Pigmented is darker than their skin color).

Pattern (P)

Each metaphoric term corresponds to a pattern in DEVO, and as in EVO, a pattern is a composite of all of the visual elements (e.g., Pigment Network contains interconnecting pigmented lines surrounding large hypopigmented circles). Further, since a pattern can itself contain one or more patterns, within DEVO we grouped together some similar metaphoric terms into higher level patterns. These higher level patterns include the Blotch Pattern (Blotch Regular, Blotch Irregular), Globules Pattern (Globules Regular, Cobblestones, Rim of Brown Globules,Globules Irregular), Dots Pattern (Dots Regular, Dots Irregular), Streaks Pattern (Radial Streaming, Pseudopods, Branched Streaks), Shiny White Structures (Shiny White Streaks, Shiny White Blotches & Strands, Rosettes), and Network Pattern (Pigment Network, Negative Network).

Spatial Pattern (SP)

We extended the Spatial Pattern concept from EVO to include Polygon Formation, Square Formation, and Leaflike Pattern, when various shapes taken in combination may form a larger shape-like structure within a pattern. For instance, the Rosettes pattern contains bright white circles arranged to form a square. Of note, there are no actual squares present in the pattern, only circles, so this is distinct the Shape concept.

Stroke (SK)

For both EVO and DEVO, the Stroke concept has the object property hasCategoryWidth for Thin and Thick Widths. In DEVO, we also added Uniform and Variable Widths to describe the lines in Typical and Atypical Pigment Networks, respectively.

Spatial Relationship (SR).

We extended the Spatial Relationship concept from EVO to include Interconnecting, Incomplete Connection, Non-interconnecting, Parallel, Orthogonal, Radial, Clustered, Distributed, Symmetrical, Asymmetrical, Uniform, and Variable Spatial Relationships in DEVO. Shapes may use the object property hasSpatialRelationship to describe their spatial layout within the pattern. Additional object properties that describe the spatial relationships between two shapes include spatialCover and spatialMeet from EVO, and spatialSurround (new in DEVO).

Size (SZ)

We extended the Size concept from EVO to include Small, Large, Long, Uniform, and Variable Sizes in DEVO. Small Size was sufficient to describe both small circles and short lines, but we defined Large Size and Long Size separately to differentiate between large circles and elongated circles, or ellipses (there were no instances of large or long lines).

Relative Position (RP)

This was a new concept created for DEVO which included the subclasses Center, Off-Center, and Periphery. Shapes may use the object property hasRelativePosition to describe their relative location within the skin lesion.

Body Position (BP)

This was a new concept created for DEVO which included the subclasses Face and Volar (palm of the hand or sole of the foot). Patterns may use the object property locatedAtBodyPosition to describe their location on the body, as some patterns only appear on certain areas (if unspecified, the lesion may be on any part of the body). However, it is important to note that this would be background information that is already known, as it is difficult to determine body position from an image of a small area of skin.

**Fig. 5:**
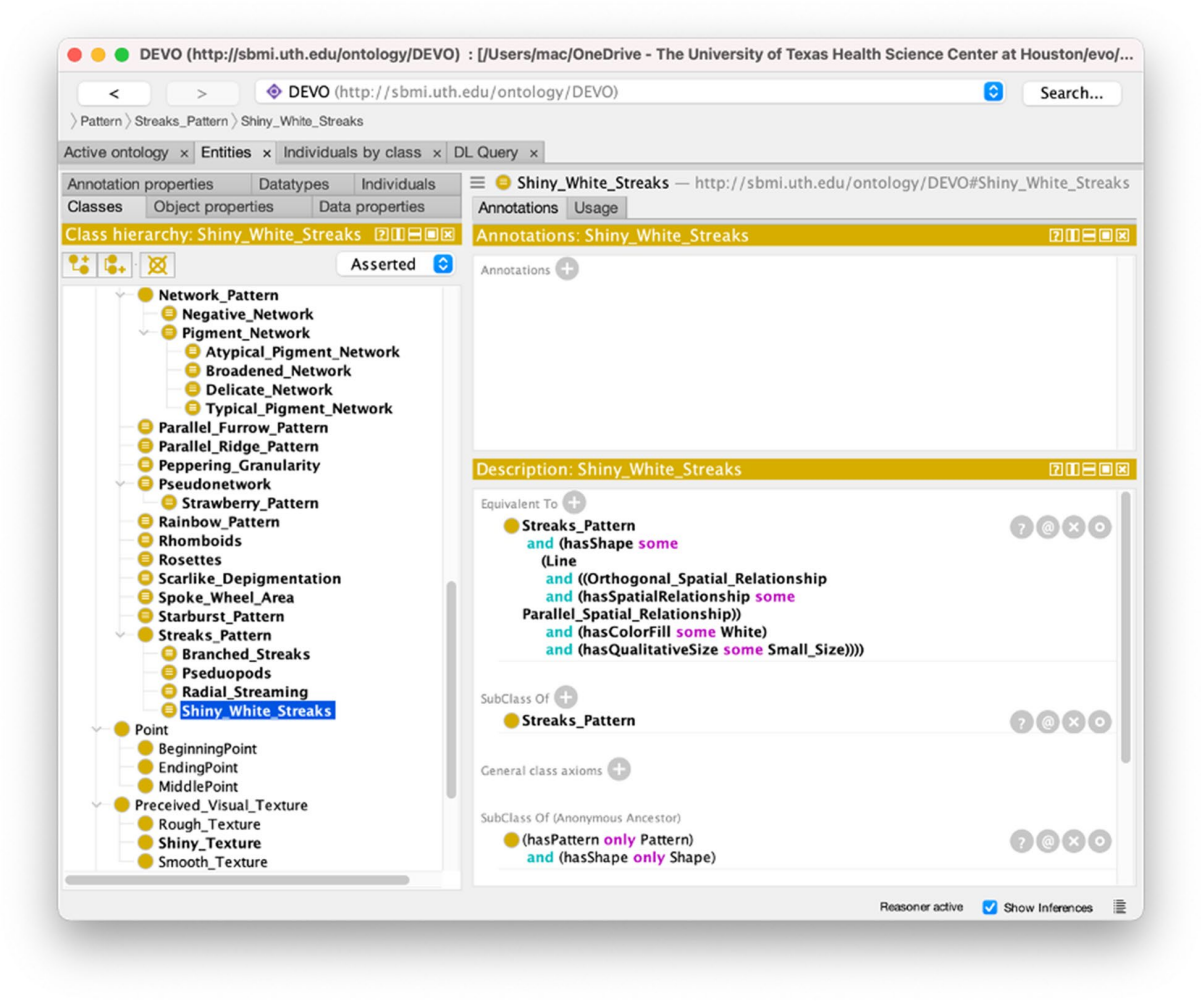
Protégé screenshot for DEVO

DEVO was coded using OWL2 with no logical inconsistencies revealed through the reasoner (FaCT +  + 1.6.5). Figure 5 shows a screenshot of the ontology viewed in Protégé.

### Evaluation of DEVO

We evaluated DEVO’s quality using the command line version of OntoKeeper, that leverages the semiotic ontology metrics that measures specific traits of an ontology that are rooted in semiotic theory – syntactic, semantic, and pragmatic. We collected a set of ontologies from NCBO Bioportal to compare qualities of DEVO with ontologies that are of the similar domain. We used Dermatology Lexicon (DL), Human Dermatological Disease Ontology (HDDO), and the Skin Physiology Ontology (SPO), and computed their qualities with the semiotic framework of ontology evaluation suite.

We have also recruited three domain experts to evaluate the consistency, complexity, and future application of our ontology. We provided two sections for them to evaluate. The first section included 48 short answer questions to evaluate the accuracy of our ontology. We provided the definitions of 48 metaphoric terms from Table 2 of Kittler et al.’s third consensus paper (Supplementary File 1) and our own ontology language that also defined those terms. The questions asked the annotator whether they thought the defined ontology language correctly conveyed the meaning of those terms. We developed two free response questions in the second section to evaluate the utility of our ontology: (1) Are the definitions provided by DEVO clearer, equally clear, or less clear than existing definitions (online/textbooks)? (2) Are there terms you use in your clinical practice that are missing from DEVO? If so, what are they?

## Results

### Evaluation of EVO

After completing the review, we re-coded the answers numerically: "X" and "N" to "0" and "Y" to "1." For the former, we presumed that if the axiom was not clear, it should be counted as a negative. We computed a percentage of "1"s for each evaluator (0.90, 0.63, and 0.92). We also computed the percentage pairwise agreement among the evaluators which yielded 72.9%, indicating there is some majority agreement of the review by our evaluators. The average agreement of the veracity of the EVO computed to be 0.82 (average of 0.90, 0.63, and 0.92), signifying the amount of accurate domain knowledge of visualizations encoded in EVO.

We collected statements that had two or three combinations of "X" (Don't Know) or "N" (No agreement to the veracity of the statement). There were 25 statements that fit this criterion, and we examined the reasons for their contention. One statement conflicted with our definition (Definition 3) that related to *hasPattern* having only one *Pattern*. According to Definition 3, *hasPattern* can include more than one *Pattern*, and we modified the functional characteristic of *hasPattern* to reflect this.

For 12 of the statements, it was revealed that the semantic definitions for the *Polygons* required a more accurate description of the number of points (e.g., “every decagon is something that has at most 10 points …”). The “most” part was redefined as "exactly"—hasPoints max 10 Points to hasPoints exactly 10 Points. Another 10 statements needed clarification on the data property domain to define that it was a numerical value and not a literal (e.g., "everything is something that has at most 1 stroke-width that is Literal"). This was modified in the ontology to reflect a numerical value.

### Evaluation of DEVO

DEVO was authored using Protégé with imports of EVO and SKOS (Simple Knowledge Organization System) to support the construction of the ontology. We used SKOS to facilitate alternative labels and annotations, such as alternative or preferred labels of the concepts. At the time of publication, DEVO contains 1047 classes, 47 object properties and 16 data properties. Both DEVO and its sibling EVO is hosted on Github [29].

The semiotic evaluation results are presented in Table 1, along with side-by-side comparison with similar ontologies of the same domain—Dermatology Lexicon (DL), Human Dermatological Disease Ontology (HDDO), and the Skin Physiology Ontology (SPO). Z-scores were also computed across the values to further assess the results.

**Table 1:** Semiotic evaluation of DEVO with similar ontologies related to dermatology

		**DEVO**	**DL**	**HDDO**	**SPO**
**Syntactic**		0.782	0.417	0.577	0.692
	*Richness*	*0.564*	*0.333*	*0.154*	*0.385*
	*Lawfulness*	*1*	*0.501*	*1*	*1*
**Semantic**		0.986	0.669	0.946	0.848
	*Clarity*	*0.988*	*0.999*	*0.998*	*0.983*
	*Consistency*	*1*	*0.0004*	*0.934*	*0.981*
	*Interpretability*	*0.964*	*1*	*0.901*	*0.575*
**Pragmatic**		0.02	0.22	0.065	0.006
	*Comprehensiveness*	*0.02*	*0.22*	*0.065*	*0.006*
**OVERALL SCORE**		**0.597**	**0.436**	**0.53**	**0.516**

For the expert evaluation, we summarized three annotators’ responses regarding 48 ontology-definition questions as follows. One annotator found 12 (25%) of them not equal to the original definition, and another annotator found 5 (10.4%) of them not exactly accurate. The last annotator agreed with all the 48 terms. We refined DEVO based on their comments and the second round of evaluations reached 100% agreement among the three experts. When asked about the utility/accuracy of the ontology, two annotators found our definitions equally clear to the existing resources, and one annotator stated that it was important to include an example picture to truly learn the terms.

## Discussion

We made the necessary revisions to EVO based on our assessment. Overall, despite some erroneous statements, most of the ontology's current domain knowledge models aspects of visualization domain knowledge. We presume there are more expansive concept definitions that can further elaborate the description of visualization elements. For a first iteration, we envision expanding the domain space of the ontology as EVO evolves.

### Interpretation of results

The evaluation metrics for the qualities of DEVO indicated that the ontology is of higher quality when considering syntactic features, semantics, and coverage of the ontology. However, with the latter, some improvements need to be made as it fared worse compared to other ontologies of the same domain. The *overall score* of DEVO computed to 0.597 (*z* = 1.17) indicating a better than average overall score than DL (0.436), HDDO (0.53), and SPO (0.516). The *syntactic* score, which specifically measures the utilization of machine-level syntax and minimal logical consistency with the use of syntax, was also better than average with a score of 0.782 (*z* = 1.05). This is due to the richness score (0.564, z-score = 1.22), which indicates that DEVO utilizes an extensive and diverse set of OWL2 expressions in the ontology compared to the others. The weakest aspect of DEVO comes from the *pragmatic/comprehensiveness* score that assesses the domain coverage based on the amount of ontology elements (instances, classes, object and data properties). The computed score for this was 0.02, z-score = -0.589. This is reasonable considering dermoscopy is a subfield of dermatology and we built our ontology to standardize 48 specific metaphoric terms in Supplementary File 1. The Dermatology Lexicon had the highest *pragmatic/comprehensiveness* score (0.22, z-score = 1.45) due to its large coverage of terms. Nonetheless, DEVO’s overall score for quality is better than similar dermoscopic and skin ontologies.

Three annotators’ feedback generated great insights of how we can further improve DEVO. One thing we noticed is that some classes from EVO did not adapt well to DEVO. One term that all three annotators commented on is “Stroke.” They found it hard to understand and an inaccurate definition of dermoscopic lesions, because a well-demarcated lesion is different from having a border or outline. Furthermore, each annotator had their own interpretation of the dermoscopic terms, and there were some slight differences between them. During the evaluation, we found that sometimes it was not our ontology definition that the annotators disagreed with, but rather the original definition itself. For example, a grey-brown color is an important property of “Annular Granular Pattern,” which was not mentioned in the original definition. Another reason some evaluators disagreed with terms in the ontology was because some details that were not directly related to the dermoscopic features are still important to include. For example, the “Rainbow Pattern” is detected under polarized light dermoscopy. However, as this is not a visual element, we decided to include it as an annotation within our ontology. The annotators also helped us better understand some terms; for example, “Ridge Pattern” was defined as lines that have variable and distributed spatial relationships in the first version. Our annotators pointed it out that “Skin Lines” would be more accurate.

The reviewers also provided great suggestions for consistency, recommending that if we have defined a certain category for one term, then we should include it as a feature for all the other terms. For example, if there is a defined color category, all the related terms should have a color definition. We adopted a bottom-up method to summarize the metaphoric terms from the third consensus paper, but there are still blanks to fill. We have modified DEVO to address all of these above suggestions and will continue to refine it moving forward.

### Limitations

Even though we have extended the Color concept in DEVO to include more dermoscopic-related colors, such as Non-pigmented Color, Hypopigmented Color, Pigmented Color, and Dark Color, one thing that is missing from DEVO is the spectrum of skin color. Some color definitions vary under different skin tones. For example, the “Blue White Veil” pattern contains white scar-like depigmentation that does not strictly look white on a dark skin tone. We plan to add the Fitzpatrick scale to further incorporate those situations.

Additionally, the coverage of DEVO is limited to the 48 terms summarized in Kittler’s paper (Supplementary File 1). The table does not list certain features, such as color or location, for each term. Also, the scope of the terms included in the paper may not be comprehensive enough. In our free-response question, when asked about the coverage of DEVO, one annotator did not identify any terms from their clinical practice missing from DEVO. Another annotator provided one term “Vessels as Dots,” which is useful for inflammatory lesions but missing from DEVO. The third annotator found vessel patterns missing in general, such as Arborizing, Glomerular, Hairpin, Crown, Serpiginous, Comma-Shaped, Dotted, Serpentine or Irregular Linear, Polymorphous, Corkscrew, etc. More dermoscopic terms are used in clinical practice and should be integrated in the ontology. While this ontology is a first version release, we anticipate future expansions to DEVO will steadily improve its coverage.

### Future direction

DEVO not only encodes basic concepts and relations among imaging features and skin diseases in a machine-interpretable way, but it also can be used to accelerate the sharing of a common understanding of information among domain experts or even software agents. This can enable the reuse of domain knowledge in a consistent manner, which would enhance patient care. It can also make it easier for new users (medical students, dermatology residents, or even dermatologists) to learn basic concepts in a short time.

There are some functions we could add on top of the ontology, such as query searching. Users could simply enter the type of features they want to find, such as “dark circles” or “white lines” to find all similar features and their relationships with skin diseases. Alternatively, they could search the ontology for features associated with specific diseases, such as “What patterns are suggestive of melanoma?”

Our next step is to integrate possible diagnoses with dermoscopic features so that the computer can return suggested skin diseases based on the detected features. We believe our DEVO knowledge can help with artificial intelligence in disease diagnosis. Convolutional neural networks have demonstrated great potential in clinical image classification. Once we have the knowledge graph converted from DEVO, we can use a methodology such as graphing convolutional networks to better help computers recognize the dermoscopic features within an image.

## Conclusion

In this paper we discuss the creation of two ontologies: the Elements of Visuals Ontology (EVO), an lower-level ontology to help decompose the visual elements of physical entities, and the Dermoscopy Elements of Visuals Ontology (DEVO), a domain ontology that harnesses EVO to formalize the definitions of dermoscopic metaphoric terms. These ontologies encode knowledge in a way that machines can understand, and they may facilitate automation of large-scale computer analysis for dermoscopy. A well-defined, standardized dermoscopic vocabulary would also enhance trainee education and patient care. The future goal is to generate responses to queries about dermoscopic features and integrate these features with diagnosis of skin diseases. Additionally, our ontologies could enable machine-based agents to store knowledge and perform basic machine intelligence.

## Supplementary Information

**Additional file 1.** List of definitions of metaphoric terminologies by Harald Kittler et al.

## Data Availability

Our ontology can be found on GitHub at Dermoscopy Elements of Visuals Ontology (DEVO): https://github.com/UTHealth-Ontology/DEVO

## Abbreviations

BP: Body position
C: Colors
DL: Dermatology Lexicon
DEVO: Dermoscopy Elements of Visuals Ontology
EVO: Elements of Visuals Ontology
HDDO: Human Dermatological Disease Ontology
P: Patterns
PATO: Phenotype And Trait Ontology
PH: Paths
PT: Points
RP: Relative position
S: Shapes
SP: Spatial patterns
SK: Strokes
SR: Spatial relationships
SZ: Sizes
SPO: Skin Physiology Ontology
SKOS: Simple Knowledge Organization System
SVG: Scalable Vector Graphic
T: Textures
VE: Visual Elements
