# Supplementary material for: DEVO: an ontology to assist with dermoscopic feature standardization
Source: BMC Med Inform Decis Mak. 2023 Aug 18;23(Suppl 1):162. doi: 10.1186/s12911-023-02251-y (PMC10436380; doi:10.1186/s12911-023-02251-y)
Supplement: Supplementary file 1 — Additional file 1. List of definitions of metaphoric terminologies by Harald Kittler et al. [file 12911_2023_2251_MOESM1_ESM.docx]

Appendix I: ﻿List ﻿of definitions of metaphoric terminologies by ﻿Harald Kittler et al.

| **Metaphoric term** | **Definition** |
| --- | --- |
| Pigment network | Gridlike pattern consisting of interconnecting pigmented lines surrounding hypopigmented holes |
| Typical pigment network | Network with minimal variability in the color, thickness, and spacing of the lines; symmetrically distributed |
| Delicate network | Light-brown, thin network lines |
| Atypical pigment network | Network with increased variability in the color, thickness, and spacing of the lines of the network; asymmetrically distributed; gray color |
| Broadened network | Widening of the network lines |
| Negative network | Serpiginous interconnecting broadened hypopigmented lines that surround elongated and curvilinear globules |
| Shiny white streaks | Short discrete white lines oriented parallel and orthogonal (perpendicular) to each other seen only under polarized dermoscopy |
| Shiny white blotches and strands | White structures in the form of circles, oval structures, or large structureless areas that are bright-white longer and less well-defined lines oriented parallel or distributed haphazardly, or forming blotches (shiny white clods); seen only under polarized dermoscopy |
| ﻿Rosettes | Four bright white dots or clods arranged together as a square (or a 4-leaf clover) |
| Globules Regular | Globules with minimal variability in their color, size, and shape |
| Cobblestones | Polygonal globules symmetrically distributed throughout lesion |
| Rim of brown globules | Globules distributed at the periphery of lesion |
| Globules Irregular | Globules with variability in color, size, shape, or spacing and distributed in an asymmetric fashion |
| Dots Regular | Dots clustered at the center of the lesion, or located on the network lines (also called target network) |
| Dots Irregular | Any distribution of dots other than dots as described for regular dots |
| (Streaks) Radial streaming | Radial linear extensions at the lesion edge |
| Pseudopods | Bulbous and often kinked projections seen at the lesion edge, either directly associated with a network or solid tumor border |
| Branched streaks | Broadened or widened network with broken lines and incomplete connections |
| Starburst pattern | This pattern consists of peripheral globules, pseudopods, or streaks (or a combination of them), located around the entire perimeter of the lesion |
| Homogeneous pattern | A pattern lacking any definable pigment structures, structureless pattern |
| Cerebriform pattern | Thick curved lines created by gyri and keratin-filled sulci; these gyri and sulci coalesce forming a brainlike appearance pattern |
| Fingerprint pattern | Light-brown thin curved lines that do not interconnect to form a network; these tend to be linear to curvilinear; they correspond to small and thin gyri |
| Rainbow pattern | ﻿Circumscribed structureless areas displaying colors of the whole spectrum of visible light |
| Strawberry pattern | Reddish pseudonetwork (erythema and wavy fine vessels) around hair follicle openings that are accentuated with a white halo appearance |
| Blotch Regular | One blotch within center of lesion and surrounded by network |
| Blotch Irregular | More than one blotch or a blotch that is located off center |
| Peppering/granularity (Regression structures) | ﻿Consists of fine dots with a blue-gray color |
| ﻿Scarlike depigmentation | Area of white that is whiter than surrounding normal-appearing skin (true scarring); it should not be confused with hypopigmentation or depigmentation caused by simple loss of melanin; shiny white structures and blood vessels are not seen in areas of regression |
| Blue whitish veil | An irregular shaped blotch of blue hue with an overlying whitish ground- glass haze |
| Angulated lines (polygons, zig-zag pattern) | Gray-brown lines that are connected at an angle or coalescing to form polygons |
| Central white patch | Central white structureless area |
| Leaflike areas | Brown to gray/blue discrete linear or bulbous structures coalescing at a common off-center base creating structures that resemble a leaflike pattern |
| Spoke wheel area | Well-circumscribed radial projections, usually light brown but sometimes blue or gray in color meeting at a central darker clod that has a dark brown, black, or blue color |
| Blue gray ovoid nest | Well-circumscribed ovoid structures with confluent or near confluent blue-gray pigmentation |
| Milia-like cyst (cloudy or starry) | White to yellowish round opalescent structures corresponding to intraepidermal cysts; when they are small and bright they are called starry; when they are larger and less bright they are called cloudy |
| Comedo-like opening | Round to oval keratin-filled clefts |
| Crypts | Keratin-filled invaginations that are larger than comedo-like openings |
| Moth-eaten border | Border with concave or sharp punched-out invaginations |
| Milky-red areas | Milky-white appearance or pinkish structureless areas (strawberry and ice creamelike), consisting a red vascular blush with no specific distinguishable vessels |
| (Facial skin) Annular granular pattern | Dots and structureless areas arranged around follicle openings (and involving adnexal opening) |
| (Facial skin)Rhomboids | Gray-brown angulated lines forming a polygonal shape around adnexal ostial openings |
| (Facial skin) Pseudonetwork | A structureless pigment area interrupted by nonpigmented adnexal openings |
| (Facial skin) ﻿Asymmetric pigmented follicular openings | Pigment associated with adnexal opening that does not uniformly surround the entire opening or curved (or crescent-shaped) pigment lines partially surrounding adnexal openings |
| (Volar skin) ﻿Parallel furrow pattern | Volar pigmentation forming solid or dotted lines, parallel, thin, on the furrows (sulci superficiales or invaginations in dermatoglyphics); the lines are occasionally doubled, each line is beside the furrows |
| (Volar skin) ﻿Parallel ﻿ridge pattern | ﻿Volar pigmentation forming lines, parallel, diffuse, and irregular, along the ridges or cristae superficiales (raised portion of the dermatoglyphics) |
| (Volar skin) ﻿﻿Latticelike pattern | Volar pigmentation forming thin lines, parallel on the furrow or sulci superficialis (invaginations in dermatoglyphics) and crossing perpendicular on the ridges |
| (Volar skin) ﻿﻿Fibrillar pattern | Linear pigmented filamentous lines of similar length with one end at the furrows and oriented at a certain angle to the furrows and crossing the ridges |
